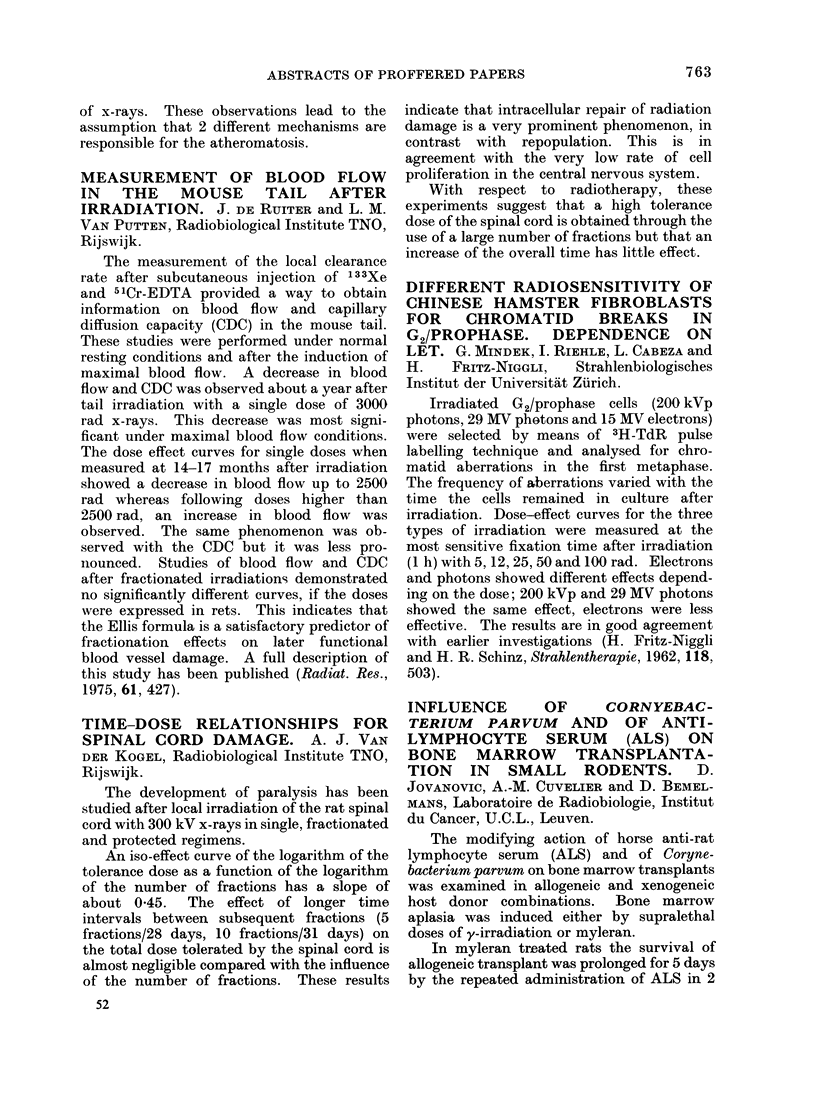# Proceedings: Time-dose relationships for spinal cord damage.

**DOI:** 10.1038/bjc.1975.329

**Published:** 1975-12

**Authors:** A. J. Van der Kogel


**TIME–DOSE RELATIONSHIPS FOR SPINAL CORD DAMAGE.** A. J. Van der Kogel, Radiobiological Institute TNO, Rijswijk.

The development of paralysis has been studied after local irradiation of the rat spinal cord with 300 kV x-rays in single, fractionated and protected regimens.

An iso-effect curve of the logarithm of the tolerance dose as a function of the logarithm of the number of fractions has a slope of about 0·45. The effect of longer time intervals between subsequent fractions (5 fractions/28 days, 10 fractions/31 days) on the total dose tolerated by the spinal cord is almost negligible compared with the influence of the number of fractions. These results indicate that intracellular repair of radiation damage is a very prominent phenomenon, in contrast with repopulation. This is in agreement with the very low rate of cell proliferation in the central nervous system.

With respect to radiotherapy, these experiments suggest that a high tolerance dose of the spinal cord is obtained through the use of a large number of fractions but that an increase of the overall time has little effect.